# Precision Healthcare and Interventions in Hereditary Breast and Ovarian Cancer and Lynch Syndrome

**DOI:** 10.3390/cancers15235601

**Published:** 2023-11-27

**Authors:** Maria C. Katapodi

**Affiliations:** Department of Clinical Research, Faculty of Medicine, University of Basel, Missionstrasse 64, 4055 Basel, Switzerland; maria.katapodi@unibas.ch

Precision health refers to personalized healthcare that combines genetic and genomic sequence, protein, metabolite, and microbiome information (collectively known as “omics” information) with lifestyle, social, economic, cultural, and environmental influences to help individuals achieve optimal health and well-being [1,2]. The goal of precision healthcare is to stratify patients and improve diagnosis and treatment based on classification strategies that enable the matching of interventions to underlying mechanisms of disease in subgroups of patients. Precision public health evolved from personalized medicine and is a multidisciplinary field that uses genomics, big data, and analytic methods that are based on artificial intelligence and can handle large quantities and diverse types of data in order to predict health risks and outcomes and to improve health at the population level [3,4].

The need to provide precision healthcare to hereditary breast and ovarian cancer (HBOC) and Lynch Syndrome (LS) patients and their families has long been recognized and has been supported with efforts at national and international levels [5,6,7]. The Centers for Disease Control and Prevention (CDC) Office of Public Health Genomics identified HBOC and LS as Tier 1 genomic applications, where empirical evidence supports early detection and precision public health interventions [8]. The field is quickly evolving, with improvements in care based on specific actionable variants in the germline. However, there are significant knowledge gaps regarding the care continuum for these two syndromes, including identifying individuals at risk of carrying the familial pathogenic variant, estimating the risk for primary and metachronous cancers, medical and lifestyle interventions that may lower cancer risks, psychosocial care needs of individuals and families, and reaching underserved populations with these two syndromes [9,10].

In this Special Issue of *Cancers*, titled “Precision Healthcare and Interventions in Hereditary Breast and Ovarian Cancer and Lynch Syndrome”, four reviews and three original research articles highlight recent trends, updates, and progress in caring for HBOC and LS individuals and families. The papers cover precision healthcare from prevention and early detection, treatment of disease, and psychosocial care of individuals carrying HBOC- and LS-associated variants and their biological relatives.

Vicente and colleagues review evidence supporting the prevalence of *BRCA1* and *BRCA2* founder pathogenic variants within the Portuguese population. Their conclusions have implications for the development of genetic testing panels, the cost-effectiveness of cascade testing among biological relatives, and the accuracy of risk prediction models that are commonly used in clinical practice to aid medical decision making in prevention, early detection, and cancer surveillance.

Two reviews by Gambini and colleagues and by Cassar and colleagues focus on the molecular mechanisms of carcinogenesis in LS and in ovarian cancer, respectively, and provide insights into opportunities for future therapeutic interventions. Gambini and colleagues present recent guidelines regarding early detection in LS patients. Regarding primary prevention, the authors focus on the comparative advantages of chemoprevention with aspirin, other non-steroidal anti-inflammatory drugs, and progestins over risk-reducing surgery, and highlight concerns over limited or conflicting evidence regarding some of the chemoprevention agents. Cassar and colleagues focus on the role of regulatory T cells in the development of ovarian cancer and review the role of these cells in pathophysiological mechanisms regulating the tumor microenvironment, angiogenesis, metastasis, drug resistance, and tumor immunity. Both reviews by Gambini and colleagues and by Cassar and colleagues emphasize the possibilities of immunotherapies that harness frameshift peptide effector T-cell responses, immunocheckpoint inhibitors, epigenetic drugs, and combinations of immunotherapies with regulatory T-cell-targeting drugs as promising new therapeutic pathways.

Bernsetin-Molho and colleagues review recommendations for managing individuals with HBOC, focusing especially on those who have not developed cancer, and for whom primary cancer prevention and early detection are of pivotal importance. The authors reviewed 15 guidelines published by governmental and professional bodies in the US, Europe, and Australia, and other countries around the world. They point out inconsistencies, including conflicting or limited evidence in areas of modifiable risk factors, such as the age of first live birth, the use of oral contraception, and the use of tamoxifen for chemoprevention. For early detection, they point out the lack of consensus regarding the optimal surveillance and risk management of younger (<30 years old) and older (>60 years old) individuals with HBOC-associated pathogenic variants, and the conflicting or limited evidence regarding the value of biomarkers such as CA-125 and screening for pancreatic cancer. Finally, they review evidence regarding effects of in vitro fertilization and pre-implantation genetic testing diagnosis, and suggest that consistent evidence shows that there is no association between in vitro fertilization and the risk of breast and ovarian cancer.

Hesse-Biber and colleagues focused on the psychosocial management of women with HBOC and conducted a mixed-methods study with women carrying a pathogenic *BRCA1* or/and *BRCA2* variant. Their study included findings of a survey with *n* = 505 participants and in-depth interviews with a subsample of *n* = 40 participants. The study focused on childbearing practices before and after knowing one’s *BRCA* status and on decision-making processes and self-conceptualization. The study found that most women of reproductive age who already had children opted not to have more children, while younger women who did not have children were likely to have children after risk-reducing surgery. Regardless of their childbearing practices, many participants felt significantly vulnerable and stigmatized, especially if they had already developed cancer. The sense of vulnerability did not diminish over time because the focus shifted from oneself to one’s family. The study sheds light on a relatively unexplored topic and identifies unmet needs of these women for ongoing care and support.

Finally, the studies of Pedrazzani and colleagues and Sarki and colleagues focus on family-mediated communication of genetic testing results and the impact on cascade testing of biological relatives. Pedrazzani and colleagues examined genetic literacy and the flow of information from carriers of pathogenic variants who had genetic counseling to their biological relatives who did not have counseling. The study combined data from three studies conducted in the U.S. and in Switzerland over 10 years with a pooled sample of *n* = 1933 participants from *n* = 518 family units harboring HBOC-associated variants. The study found that, although genetic literacy was higher among participants who had counselling, some risk factors were poorly understood, especially among those that had genetic counseling more than 5 years ago, had fewer years of formal education, and did not have a pronounced family history of cancer. Sarki and colleagues provided strong evidence for the potential impact of cascade testing as a precision public health intervention, as their study found that, from 304 individuals with HBOC- or LS-associated variants and 115 of their relatives, on average 10 biological relatives per participant were potentially eligible for cascade genetic testing. However, only two out of three individuals with a pathogenic variant wanted to invite their relatives to the cohort, and about 50% indicated a preference for family-mediated communication of testing results, possibly with the assistance of digital technology. Both studies emphasize the importance of the family environment as a means to facilitate better management of HBOC and LS and the implementation of cascade testing as a precision public health intervention.

In conclusion, technological advances and the growing application of precision healthcare and precision public health interventions have increased our knowledge of HBOC and LS and offer pathways for novel and personalized therapeutic opportunities. Personalized therapeutic opportunities will be most advantageous for individuals diagnosed with cancers associated with high morbidity and mortality, such as ovarian, pancreatic, and bile duct cancers. However, the translation of this knowledge into concrete and consistent prevention and early detection guidelines is lagging behind, due, to limited evidence from large epidemiological studies with comprehensive assessments of genetic and genomic, socioeconomic, cultural, and health behavioral data. There is even less evidence regarding the translation of this knowledge into the equitable psychosocial care of these individuals and families. This Special Issue helped identify areas where more evidence, from high-quality studies, is needed.
